# Approaches to Invasive Fungal Diseases in Paediatric Cancer Centres: An Analysis of Current Practices and Challenges in Germany, Austria and Switzerland

**DOI:** 10.1111/myc.70074

**Published:** 2025-06-14

**Authors:** Danila Seidel, Zoi Dorothea Pana, Daniel Ebrahimi‐Fakhari, Sarina K. Butzer, Katrin Mehler, Ilana Reinhold, Arne Simon, Christian Dohna‐Schwake, Ines Mack, Nicole Bodmer, Tim Niehues, Alexander Claviez, Alfred Längler, Alfred Leipold, Aram Prokop, Bastian Brummel, Beate Winkler, Bernd Gruhn, Carl Friedrich Classen, Carsten Friedrich, Christa Koenig, Christian Flotho, Fiona Poyer, Freimut Schilling, Gabriele Calaminus, Geeke Sieben, Georg C. Schwabe, Harald Reinhard, Heiko‐Manuel Teltschik, Heinz Hengartner, Jana Stursberg, Jeanette Greiner, Johann Greil, Jörg Leyh, Jörn‐Sven Kühl, Karoline Ehlert, Konrad Bochennek, Marius Rohde, Martin Demmert, Martina Stiefel, Matthias Eyrich, Meinolf Siepermann, Michael Frühwald, Michaela Döring, Michaela Nathrath, Milen Minkov, Monika Streiter, Neil Jones, Nora Naumann‐Bartsch, Norbert Jorch, Olaf Beck, Rita Beier, Roman Crazzolara, Silke Kietz, Simon Vieth, Stefan Fröhling, Stephan Lobitz, Sujal Ghosh, Tanja C. Vallée, Thilo Müller, Thomas Wiesel, Tobias Däbritz, Udo Kontny, Uwe Thiel, Volker Strenger, Wolfgang R. Eberl, Oliver A. Cornely, Andreas H. Groll, Thomas Lehrnbecher

**Affiliations:** ^1^ Institute of Translational Research, Cologne Excellence Cluster on Cellular Stress Responses in Aging‐Associated Diseases (CECAD), Faculty of Medicine and University Hospital Cologne University of Cologne Cologne Germany; ^2^ Department I of Internal Medicine, Excellence Center for Medical Mycology (ECMM), Faculty of Medicine and University Hospital Cologne University of Cologne Cologne Germany; ^3^ Medical School University of Nicosia (UNIC) Nicosia Cyprus; ^4^ Infectious Disease Research Program, Center for Bone Marrow Transplantation and Dept. of Pediatric Hematology/Oncology University Children's Hospital Münster Münster Germany; ^5^ Division of Pediatric Infectious Diseases, Department of Pediatrics, Faculty of Medicine and University Hospital Cologne University of Cologne Cologne Germany; ^6^ Division of Pediatric Hematology and Oncology, Department of Pediatrics, Faculty of Medicine and University Hospital Cologne University of Cologne Cologne Germany; ^7^ Department of Pediatric Hematology and Oncology University Medical Center of Saarland Homburg/Saar Germany; ^8^ Department of Pediatrics I, Neonatology, Pediatric Intensive Care, Pediatric Infectiology, Pediatric Neurology, University Hospital Essen University Duisburg‐Essen Essen Germany; ^9^ Department of Paediatric Infectious Diseases and Vaccinology University Children's Hospital Basel (UKBB) and University of Basel Basel Switzerland; ^10^ Department of Oncology University Children's Hospital Zurich Zurich Switzerland; ^11^ Centre for Child and Adolescent Health, Helios Klinikum Krefeld Germany; ^12^ Department of Pediatrics University Hospital Magdeburg Magdeburg Germany; ^13^ Clinic for Pediatric Oncology and Hematology, Hannover Medical School Hannover Germany; ^14^ Department of Paediatrics and Adolescent Medicine Community Hospital Herdecke Herdecke Germany; ^15^ Children's Hospital Karlsruhe Karlsruhe Germany; ^16^ Campus University Medical School Hamburg (MSH) Helios Clinic Schwerin Schwerin Germany; ^17^ Department of Pediatric Oncology and Hematology Klinikum Dortmund Dortmund Germany; ^18^ Department of Pediatric Hematology and Oncology University Medical Center Hamburg‐Eppendorf Hamburg Germany; ^19^ Department of Pediatrics Jena University Hospital Jena Germany; ^20^ Section of Pediatric Oncology and Palliative Medicine, Klinik für Kinder‐ und Jugendmedizin Universitätsmedizin Rostock Rostock Germany; ^21^ Department of Pediatrics and Pediatric Hematology/Oncology, University Children's Hospital Carl von Ossietzky Universität Oldenburg, Klinikum Oldenburg AöR Oldenburg Germany; ^22^ Pediatric Hematology/Oncology, Department of Pediatrics, Inselspital, Bern University Hospital University of Bern Bern Switzerland; ^23^ Division of Pediatric Hematology and Oncology, Department of Pediatrics and Adolescent Medicine, Faculty of Medicine, Medical Center University of Freiburg Freiburg Germany; ^24^ Department of Pediatric Hematology and Oncology, St. Anna Children's Hospital Medical University of Vienna Vienna Austria; ^25^ Pediatric Oncology‐Hematology, Children's Hospital of Central Switzerland Lucerne Switzerland; ^26^ Department of Pediatric Hematology and Oncology University Hospital Bonn Bonn Germany; ^27^ Pediatric Oncology and Hematology, University Medical Center Mannheim Mannheim Germany; ^28^ Children's Hospital Medical University Lausitz—Carl Thiem Cottbus Germany; ^29^ Department of Pediatrics Asklepios Children's Hospital Sankt Augustin Germany; ^30^ Department of Hematology and Oncology Children's Hospital Stuttgart‐Olgahospital Stuttgart Germany; ^31^ Pediatric Hospital of Eastern Switzerland St Gallen St Gallen Switzerland; ^32^ Department of Pediatrics and Adolescent Medicine Ulm University Medical Center Ulm Germany; ^33^ Pediatric Oncology‐Hematology, Children's Hopital Cantonal Hospital Aarau Aarau Switzerland; ^34^ Department of Pediatric Hematology, Oncology and Immunology University Hospital Heidelberg Heidelberg Germany; ^35^ Department of Pediatric Hematology and Oncology Klinik Hallerwiese‐Cnopfsche Kinderklinik Nürnberg Germany; ^36^ Department of Pediatric Oncology, Hematology and Hemostaseology University of Leipzig Leipzig Germany; ^37^ Department of Pediatric Hematology and Oncology University Medicine Greifswald Greifswald Germany; ^38^ Department of Pediatrics, Division of Hematology, Oncology and Hemostaseology Goethe University Frankfurt, Frankfurt/Main, Germany Frankfurt am Main Germany; ^39^ Pediatric Hematology and Oncology University Hospital Giessen Giessen Germany; ^40^ Clinic for Pediatric and Adolescent Medicine University Hospital Schleswig‐Holstein Lübeck Germany; ^41^ Department of Pediatrics Martin‐Luther‐University Halle‐Wittenberg Halle Germany; ^42^ Department of Pediatric Haematology, Oncology and Stem Cell Transplantation, University Medical Center, University Children's Hospital University of Würzburg Würzburg Germany; ^43^ Clinic for Children and Youth Medicine, Department for Pediatric Hematology/Oncology Children's Hospital Amsterdamer Straße Cologne Germany; ^44^ Swabian Children's Cancer Center, Pediatrics and Adolescent Medicine University Medical Center Augsburg Augsburg Germany; ^45^ Department of Paediatric Haematology/Oncology Children's University Hospital Tübingen Germany; ^46^ Department of Pediatrics and Children's Cancer Research Center, School of Medicine Technical University of Munich Munich Germany; ^47^ Pediatric Hematology and Oncology, Klinikum Kassel Kassel Germany; ^48^ Division of Pediatric Oncology, Department of Pediatrics and Adolescent Medicine Johannes Kepler University Linz Austria; ^49^ Clinic for Pediatrics and Adolescent Medicine, Hematology/Oncology Klinikum am Gesundbrunnen Heilbronn Heilbronn Germany; ^50^ Department of Pediatrics and Adolescent Medicine University Clinics Salzburg Austria; ^51^ Department for Pediatrics and Adolescent Medicine University Hospital Erlangen Erlangen Germany; ^52^ University Children's Hospital, Evangelisches Klinikum Bethel Bielefeld Germany; ^53^ Department of Pediatric Hematology/Oncology, Center for Pediatric and Adolescent Medicine University Medical Center of the Johannes Gutenberg‐University Mainz Mainz Germany; ^54^ Department of Pediatric Hematology and Oncology Hannover Medical School Hannover Germany; ^55^ Department of Pediatrics Medical University of Innsbruck Innsbruck Austria; ^56^ Department of Pediatric Hematology, Oncology and Stem Cell Transplantation University of Regensburg Regensburg Germany; ^57^ Department of Pediatrics University Hospital Schleswig‐Holstein Kiel Germany; ^58^ Pediatric Oncology Center Bremen‐Mitte, Professor Hess Children's Hospital Bremen Germany; ^59^ Pediatric Hematology & Oncology Gemeinschaftsklinikum Mittelrhein Koblenz Germany; ^60^ Department of Pediatric Oncology, Hematology and Clinical Immunology, Medical Faculty, University Hospital Düsseldorf Heinrich‐Heine‐University Düsseldorf Germany; ^61^ Dr. von Hauner Children's Hospital, University Hospital LMU Munich Munich Germany; ^62^ Department of Pediatric Oncology and Hematology Charité—Universitätsmedizin Berlin Berlin Germany; ^63^ Children's Hospital, Vestische Youth Hospital University of Witten/Herdecke Datteln Germany; ^64^ Department of Pediatric Hematology and Oncology, Department of Pediatrics, Faculty of Medicine and University Hospital Carl Gustav Carus Technische Universität Dresden Dresden Germany; ^65^ Division of Pediatric Hematology, Oncology and Stem Cell Transplantation University Hospital Aachen Aachen Germany; ^66^ Department of Pediatrics and Adolescent Medicine Medical University of Graz Graz Austria; ^67^ Department of Hematology and Oncology, Center for Child and Adolescent Medicine Städtisches Klinikum Braunschweig gGmbH Braunschweig Germany; ^68^ Department I of Internal Medicine, Center for Integrated Oncology Aachen Bonn Cologne Duesseldorf (CIO ABCD) and Excellence Center for Medical Mycology (ECMM), Faculty of Medicine and University Hospital Cologne University of Cologne Cologne Germany; ^69^ German Centre for Infection Research (DZIF), Partner Site Bonn‐Cologne Cologne Germany; ^70^ Clinical Trials Centre Cologne (ZKS Köln), Faculty of Medicine and University Hospital Cologne University of Cologne Cologne Germany

**Keywords:** antifungal stewardship, aspergillosis, cancer, candidaemia, children, diagnosis, management, mycoses, prophylaxis, treatment

## Abstract

**Background:**

Invasive fungal diseases (IFD) pose significant challenges in paediatric oncology. Their management is complicated by limited paediatric‐specific evidence, lack of standardised protocols and variability in resources across centres. This study assessed current practices and addressed the challenges in the prevention, diagnosis and treatment of IFDs in paediatric oncology centres across Germany, Austria and Switzerland.

**Methods:**

A questionnaire was distributed to senior paediatric oncologists in 70 paediatric oncology centres across Germany, Austria and Switzerland, gathering data on centre infrastructure, infectious disease (ID) expertise, annual cumulative IFD incidence in 2023, diagnostic tools, antifungal prophylaxis, treatment and follow‐up practices for IFD. Responses were analysed descriptively.

**Results:**

Sixty‐two centres responded, with a median of 56 (IQR 40–75) new oncological diagnoses per centre; 54.8% of centres managed allogeneic HCT patients. IFDs were reported in 88.7% of centres, with a median cumulative IFD incidence of 4.6% (IQR 3.0%–5.9%). No significant association was found between cumulative IFD incidence and the number of transplants, antifungal prophylaxis protocols and availability of ID consultation services. ID consultation was available in 58.1% of centres, with 24/7 support provided in 41.7% of these centres. Larger centres more frequently had paediatric ID specialists, ID consultation services and access to therapeutic drug monitoring.

**Conclusions:**

The observed heterogeneity in mycology expertise and IFD management strategies across centres reflects the inherent complexity of IFDs and the diagnostic and therapeutic uncertainties amid limited evidence. Strengthening oncology‐ID networks and implementing digital consultation platforms may promote high‐quality, equitable care, particularly for those with fewer in‐house resources.

## 1 Introduction

Paediatric cancer patients and allogeneic cell transplant recipients are at high risk for invasive fungal diseases (IFD). Key risk factors include prolonged chemotherapy‐induced neutropenia, corticosteroid therapy and immunosuppression used for prophylaxis or treatment of graft‐versus‐host disease (GVHD) in the transplant setting. Early diagnosis of IFD based on clinical, radiological and microbiological evaluation, including serum galactomannan screening, although its sensitivity markedly drops in children receiving mould‐active prophylaxis [1] and timely appropriate treatment including surgery where feasible are the cornerstones of effective management [2, 3]. Best practices for diagnosis and management of IFDs in children are detailed in several national and international guidelines [3, 4, 5, 6, 7]. However, it remains unclear to what extent the recommendations of these guidelines have been implemented in clinical practice within paediatric oncology. Moreover, real‐world application of EORTC/MSG definitions, criteria that are widely used in clinical practice to identify patients at risk for IFD, can be challenging, with over 40% of paediatric cases falling outside the probable or proven categories [8, 9]. Despite advances in the clinical management of patients at risk [10], new and improved diagnostic methods [11, 12], and approval of new antifungal agents [11, 13], the complexity of individual cases presents a continuous challenge hampering standardisation of management practices.

A clear framework for healthcare professionals, outlining expected practices in accordance with current clinical guidelines, is essential to ensure equitable prevention, diagnosis and management of fungal infections in paediatric patients. In this context, tools like the paed‐EQUAL score, a point‐based scoring system developed to enhance guideline adherence in the management of candidaemia in children and neonates, represent a promising step toward standardising clinical practices and improving antifungal stewardship [14]. Current variations in regional and institutional practices are due to several factors, including lack of recommendations based on robust evidence on antifungal prophylaxis for all patient populations at risk, limited clinical trials conducted in paediatric populations, differences in the availability of microbiological tests across centres and varying levels of clinical expertise [15, 16, 17]. We assessed current practices in prevention, diagnosis and management of IFDs in paediatric cancer centres in Germany, Austria and Switzerland to identify institutional and regional approaches to provide a starting point for a strategic roadmap for development of future paediatric antifungal stewardship programmes.

## 2 Methods

A survey addressing current practices in IFD prophylaxis, diagnosis and treatment was compiled in two virtual calls based on similar previous surveys in different medical settings [18, 19]. The survey was developed using the TIVIAN (Cologne, Germany) online survey platform and extensively pilot‐tested for final modifications. Paediatric oncologists from all paediatric oncology centres registered within the Society for Pediatric Oncology and Hematology (Gesellschaft für Pädiatrische Onkologie und Hämatologie, GPOH) were invited via email with the link to the survey to reply to the questionnaire in June 2024. Responses from one, preferentially senior‐level physician responsible for managing IFDs in haematological‐oncological patients ≤ 18 years were allowed for each centre.

In brief, the questionnaire covered topics such as the respondent's professional role, memberships in mycology‐related societies, participation in clinical studies and publications on mycological topics, centre characteristics such as the availability of microbiological and imaging diagnostic tools for fungal infections, paediatric oncology diagnoses and allogeneic HCT cases in 2023, the number of paediatric patients diagnosed with proven or probable fungal infections according to the current EORTC/MSG criteria [8] in 2023, the use of national and international guidelines for IFD management, the availability of standard operating procedures (SOPs) for prophylaxis, diagnosis and treatment of IFDs, and approaches to antifungal prophylaxis and treatment for pulmonary aspergillosis and candidaemia as the most common IFDs in children in the oncological setting (survey in Table S1). A reminder to complete the survey was sent after 2 months to maximise response rates. Responses were assessed for completeness and consistency, and questions were resolved with the participant via email.

Statistical analysis was performed using SPSS version 29.0.0 (SPSS Inc., Chicago, IL, USA). Responses were analysed descriptively using frequencies and percentages for categorical variables and medians with interquartile ranges for continuous variables. Mann–Whitney U Test was used to compare distribution between groups. To assess relationships between the number of invasive fungal infection cases in 2023, the annual number of paediatric patients with newly diagnosed cancer, the number of patients with HCT, established SOPs for antifungal prophylaxis and availability of ID consultation service, linear regression analyses were performed. These variables were chosen as candidate predictors for the following reasons: first, HCT is a well‐established risk factor for IFD, owing to prolonged neutropenia, GVHD and high‐dose immunosuppression in that population; second, we assumed that centres with formal SOPs for antifungal prophylaxis are likely to have more consistent risk stratification and drug dosing practices and ensure prompt prophylaxis in higher‐risk patients, which may reduce breakthrough infections; and last, routine ID consultation allows for rapid review of emerging fevers by ID specialists, earlier and potentially more elaborative diagnostic work‐up, and timely initiation or adaptation of antifungal therapy, all of which can influence the detection and eventually true incidence of IFD. For all tests, a significance level *p* ≤ 0.05 was used to determine statistical significance.

The Ethics Committee of the University Hospital of Cologne and the regional Physician's Chamber (Ethik‐Kommission der Ärztekammer Nordrhein‐Westfalen, Düsseldorf, Germany) were consulted, and both confirmed that no formal approval or authorisation was required to conduct this survey.

## 3 Results

**FIGURE 1:**
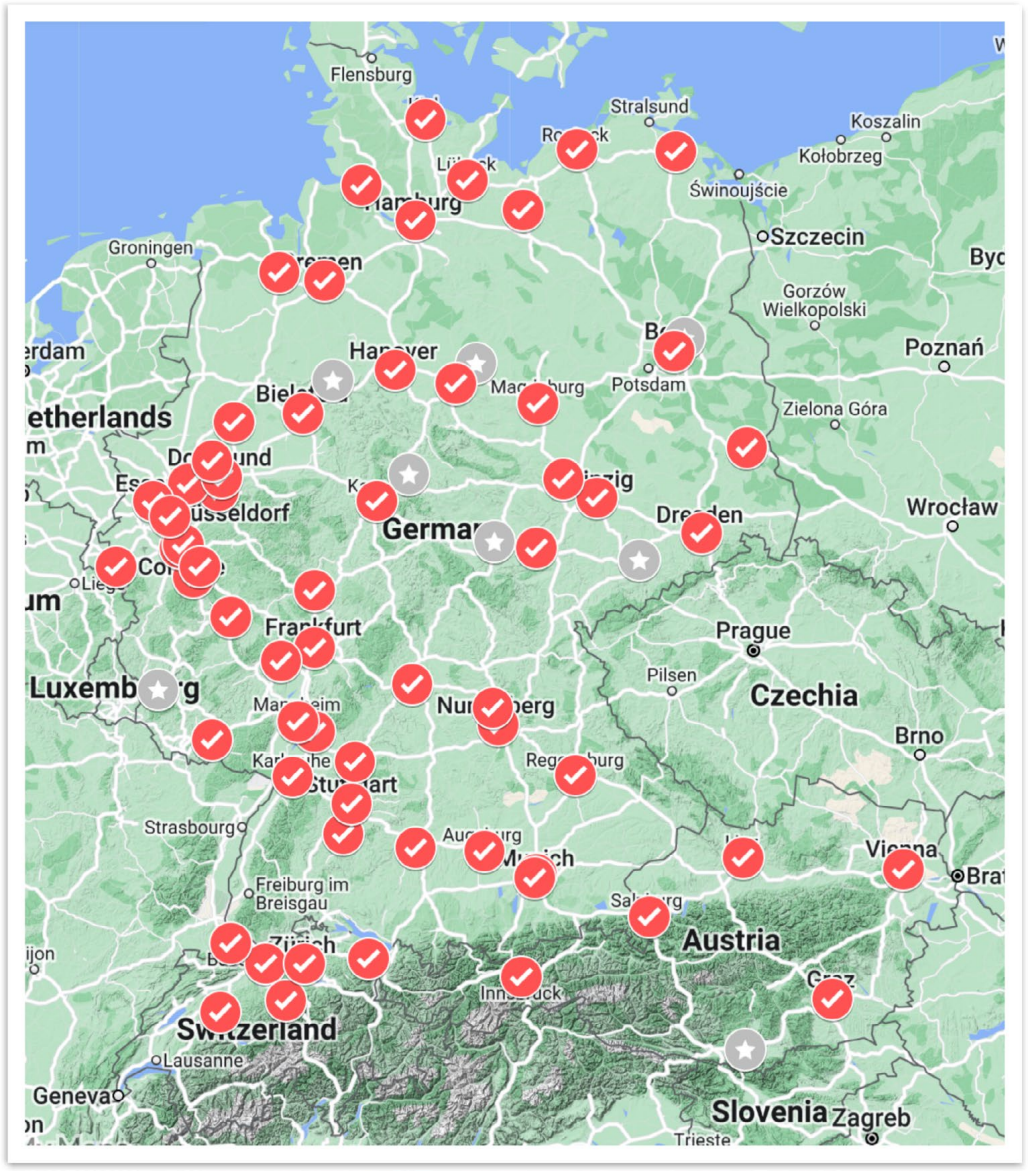
Distribution of 62 participating paediatric oncology centres (red) in Germany (51), Austria (5) and Switzerland (6) (invited centres without reply in grey). *Source:* www.google.de/maps.

Responses were available from 62 out of 70 centres (88.6%), including 51 of 58 invited German centres, five of six Austrian centres and six Swiss centres (Figure 1). In 54 centres (87.1%), respondents were department heads or senior physicians (Table 1).

**TABLE 1:** Characteristics of participating paediatric experts and oncology centres.

*N* (%)	Overall	Austria	Germany	Switzerland
**Participating paediatric oncology clinics**				
Participant female sex	62 (100.0)	5 (8.1)	51 (82.3)	6 (9.7)
Professional role	19 (30.6)	1 (20.0)	14 (27.5)	4 (66.7)
Head physician	9 (14.5)	0 (0.0)	8 (15.7)	1 (16.7)
Head physician female sex	1 (11.1)	0 (0.0)	1 (2.0)	0 (0.0)
Senior physician	45 (72.6)	5 (100.0)	36 (70.6)	4 (66.7)
Senior physician female sex	13 (28.9)	1 (20.0)	9 (17.6)	3 (50.0)
Specialist physician	8 (12.9)	0 (0.0)	7 (13.7)	1 (16.7)
Specialist physician female sex	5 (62.5)	0 (0.0)	4 (7.8)	1 (16.7)
Additional ID specialisation				
At children's hospital	36 (58.1)	2 (40.0)	28 (54.9)	6 (100.0)
At paediatric oncology centre	6 (9.7)	0 (0.0)	6 (11.8)	0 (0.0)
Membership in mycological societies	25 (40.3)	4 (80.0)	20 (39.2)	1 (16.7)
National societies	23 (37.1)	3 (60.0)	19 (37.3)	1 (16.7)
International societies	6 (9.7)	2 (40.0)	3 (5.9)	1 (16.7)
Participation in mycological studies	10 (16.1)	2 (40.0)	6 (11.8)	2 (33.3)
Participation in clinical trials	2 (3.2)	0 (0.0)	2 (3.9)	0 (0.0)
Publications on mycology topics	20 (32.3)	3 (60.0)	15 (29.4)	2 (33.3)
Department of ID	22 (35.5)	1 (20.0)	15 (29.4)	6 (100.0)
ABS expert at children's hospital	53 (85.5)	5 (100.0)	43 (84.3)	5 (83.3)
Paediatric ID consultation service	36 (58.1)	2 (40.0)	28 (54.9)	6 (100.0)
Available 24/7	15 (24.2)	1 (20.0)	8 (15.7)	6 (100.0)
Regular interdisciplinary meetings	23 (37.1)	2 (40.0)	17 (33.3)	4 (66.7)
SOPs available, any	55 (88.7)	3 (60.0)	47 (92.2)	5 (83.3)
SOP available for Px, Dx and Tx	36 (58.1)	3 (60.0)	30 (58.8)	3 (50.0)
For antifungal prophylaxis	52 (83.9)	3 (60.0)	44 (86.3)	5 (83.3)
For fungal diagnostics	37 (59.7)	3 (60.0)	31 (60.8)	3 (50.0)
For antifungal treatment	47 (75.8)	3 (60.0)	41 (80.4)	3 (50.0)
NRZMyk known	45 (72.6)	4 (80.0)	39 (76.5)	2 (33.3)
NRZMyk contacted^a^	25 (40.3)	1 (20.0)	24 (47.1)	0 (0.0)

*Note:* All cells are reported as *N* (%). Percentages for groups with *N* < 10 should be interpreted cautiously due to small denominators.

Abbreviations: Dx, diagnostics; ID, infectious disease; NRZMyk, German National Reference Center for Invasive Fungal Infections; Px, prophylaxis; SOP, standard operating procedure for managing invasive fungal infections; Tx, treatment.

^a^ Contacted for expert species identification, conducting susceptibility testing and result interpretation, or therapeutic advice on complicated cases.

The median number of new paediatric oncology diagnoses reported in 2023 was 56 (IQR 40–75, range 14–160), with a median of 13 (IQR 7–28) children who underwent allogeneic haematopoietic cell transplantation (HCT) managed in 34 (54.8%) centres.

A paediatric ID specialist was available in 58.1% (36/62) of the centres, either board‐certified (6/6 in Switzerland) or having a certified ID training (28/51 in Germany and 2/5 in Austria).

A paediatric ID department or section was available in 35.5% (22/62) of centres. Most centres (53/62, 85.5%) had access to an expert in antimicrobial stewardship overseeing the paediatric oncology department.

A paediatric ID consultation service was available in 58.1% (36/62) of centres, with 24/7 consultation availability in 41.7% (15/36). Centres with an ID consultation service had a significantly higher number of new paediatric oncological diagnoses compared to those without (median 46 vs. 68, *p* = 0.008). In Switzerland, the centres had a dedicated ID department, a 24/7 ID consultation service and regular multidisciplinary meetings. In Germany and Austria, 30% of centres had comparable services, and most operated during regular working hours from Monday to Friday. Regular interdisciplinary ID meetings were reported in 37.1% (23/62) of centres.

In 2023, proven and probable IFD were reported in 88.7% (55/62) of centres, with a median cumulative IFD incidence weighed by number of new diagnoses of 4.6% (IQR 3.0%–5.9%). Linear regression revealed the numbers of new paediatric oncology diagnoses (*B* = 0.052, 95% CI: 0.035–0.070, *p* < 0.001) predicted IFD cases, while the number of HCT patients (*p* = 0.104) and the availability of ID consultation services (*p* = 0.992) were not significant predictors.

Research in clinical mycology was conducted in 16.1% (10/62) of centres, with two centres in Germany participating in antifungal regulatory registration trials. Twenty centres (32.5%) published mycology‐related research. Membership in ID societies was reported by 35.5% (22/62) of centres, with the national German Society for Paediatric Infectious Diseases (DGPI) representing the group with the largest presence (20/62 centres, 32.2%). In Germany, memberships in the German‐speaking Mycological Society (DMykG) were reported by two centres. Additionally, memberships in international societies such as the European Confederation of Medical Mycology (ECMM), European Society for Paediatric Infectious Diseases (ESPID), Infectious Diseases Society of America (IDSA) and International Society for Human and Animal Mycology (ISHAM) were reported in 6 out of 62 centres (9.7%).

### 3.1 Diagnostic Capabilities

**FIGURE 2:**
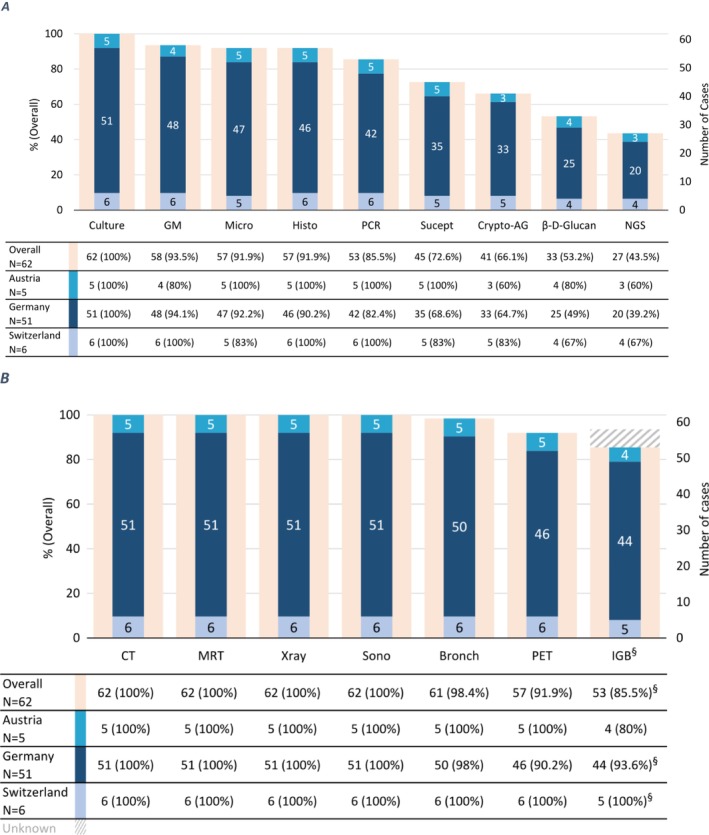
Availability of laboratory diagnostics and diagnostic imaging and procedures in 62 paediatric cancer centres. (A) Laboratory diagnostics. Crypto AG, *Cryptococcus* antigen; GM, galactomannan; Histo, histopathology; Micro, microscopy; NGS, next generation sequencing; Suscept, susceptibility testing. Colour code: % percentage (orange), *N*: number of cases (blue shades). (B) Diagnostic imaging and procedures. Bronch, bronchoscopy; CT, computed tomography; IGB, image‐guided biopsy; MRI, magnetic resonance imaging; PET, PET‐CT/PET‐MRI; Sono, sonography; Xray, radiography. ^§^IGB responses are available from 57/62 centres (47/51 Germany, 5/5 Austria, 5/6 Switzerland).

SOPs for diagnosing IFDs were available in 59.7% of centres (Table 1), with culture, direct microscopy and histopathology performed in 91.9% (51/62). Galactomannan testing in any material was available in 93.5% of centres. Access to PCR (85.5%), next‐generation sequencing (NGS) (43.5%), β‐D‐Glucan testing (53.2%) and in vitro susceptibility testing (72.6%) varied across centres (Figure 2). All centres with an ID consultation service (36/36) versus 84.6% of centres without such a service (22/26) performed galactomannan testing (*p* = 0.027). Of those centres that had susceptibility testing capabilities, 71.1% (32/45) assessed minimum inhibitory concentration (MIC) in every IFD case where culture was available, and 28.9% (13/45) assessed MICs only for certain pathogens or when resistance is suspected. In centres without in‐house susceptibility testing resources, 47.1% (8/17) never performed MIC testing, 29.4% (5/17) tested all cases and 23.5% (4/17) in certain cases.

In all centres, imaging studies including ultrasound, X‐ray, computed tomography (CT) and magnetic resonance imaging (MRI) were available, and in the vast majority also positron emission tomography (PET)‐CT or PET‐MRI and bronchoscopy (Figure 2). Fifty‐seven centres (57/62, 91.9%) provided information on image‐guided biopsy capacity; of these, 93.0% (53/57; 85.5%, 53/62) reported the ability to perform CT‐ or sonographic‐guided biopsies locally. CT imaging was available 24 h a day in 97% of centres, and MRI imaging in 80.6%.

### 3.2 Antifungal Prophylaxis and Treatment Practices

Antifungal prophylaxis and treatment followed the national paediatric AWMF guideline, and the two current international guidelines for paediatric cancer patients in all centres [3, 20, 21]. Other guidelines for IFD management, such as the global ECMM/ISHAM [22, 23, 24, 25, 26] or the IDSA clinical practice guidelines [6, 27] were used by nine and six centres, respectively.

**FIGURE 3:**
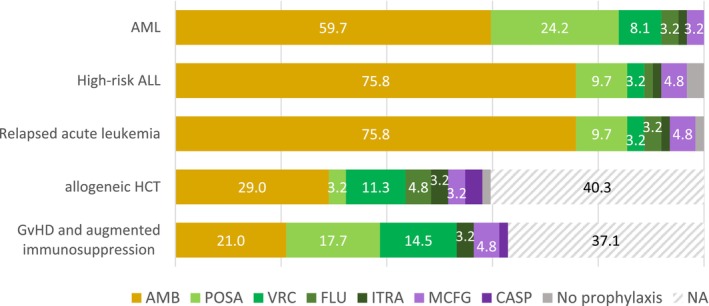
Preferred antifungal prophylaxis in different patient populations in 62 paediatric cancer centres (percent). ALL, acute lymphocytic leukaemia; AMB, liposomal amphotericin B; AML, acute myeloid leukaemia; CASP, caspofungin; FLU, fluconazole; GvHD, graft‐versus‐host disease; HCT, haematopoietic cell transplantation; ITRA, itraconazole; MCFG, micafungin; NA, not applicable; POSA, posaconazole; VRC, voriconazole.

SOPs for antifungal prophylaxis and treatment existed in 83.9% (52/62) and 75.8% (47/62) of the centres, respectively. Variability of antifungal prophylaxis and treatment strategies was noted across centres. Antifungal prophylaxis was used in all centres, with liposomal amphotericin B (L‐AMB) either daily or intermittently being the most commonly used modality across different risk groups (Figure 3). In patients with acute myeloid leukaemia (AML), high‐risk acute lymphoblastic leukaemia (ALL) and relapsed acute leukaemia, posaconazole was the second most frequently used modality, and in allogeneic HCT patients, voriconazole. In GvHD and augmented immunosuppression, centres used L‐AMB, posaconazole, or voriconazole for prophylaxis in a comparable frequency.

Antifungal treatment variability was noted. Empiric therapy was the predominant approach in 74.2% (46/62) of centres, initiated in cases of fever and neutropenia, whereas pre‐emptive strategies, relying on biomarkers and imaging results, were selected to be the preferred in the remaining.

**FIGURE 4:**
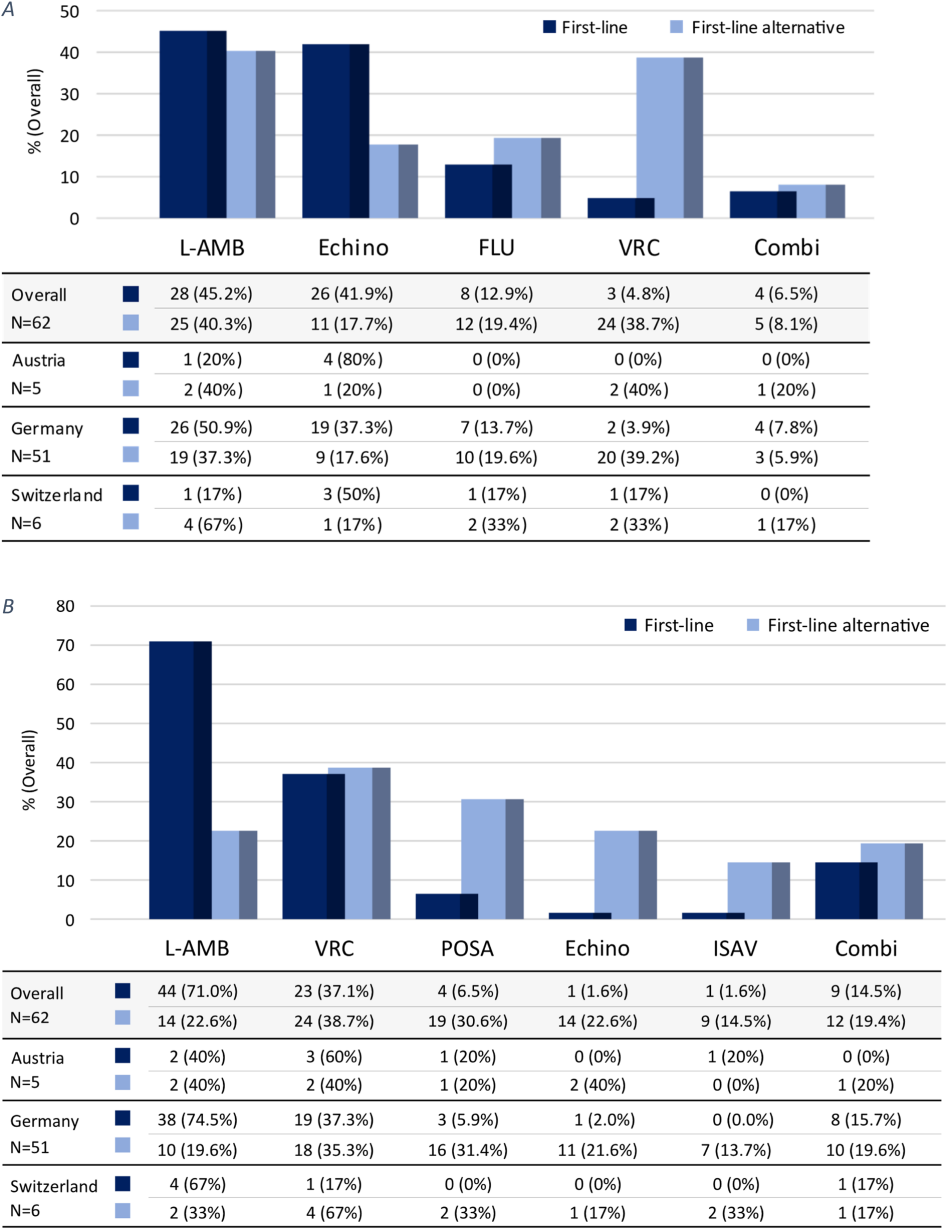
First‐line antifungal of choice and alternative for (A) candidaemia and (B) invasive pulmonary aspergillosis in 62 paediatric cancer centres. Combi, antifungal‐combination therapy; Echino, echinocandin; FLU, fluconazole; ISAV, isavuconazole; L‐AMB, liposomal amphotericin B; POSA, posaconazole; VRC, voriconazole. (A) Candidaemia (centres with multiple preferred first‐line (*N* = 4) or alternative agents (*N* = 16) were included based on their respective choices). (B) Invasive pulmonary aspergillosis (centres with multiple preferred first‐line (*N* = 14) or alternative treatments (*N* = 24) were included based on their respective choices).

In patients with candidaemia, the preferred first‐line antifungal therapy was L‐AMB in 45.1% and one of the echinocandins in 41.9% of the centres, with fluconazole being the first‐line agent in 12.9% (Figure 4A). Four centres selected more than one preferred first‐line agent and 16 centres more than one alternative agent. The first‐line echinocandin for candidaemia was proportionally higher in centres with paediatric ID consultation services compared to centres without an established ID service (52.8% vs. 26.8%, *p* = 0.067). In 24 (38.7%) centres, the treatment duration was at least 14 days after the first negative blood culture, while in the others, the treatment length was based on individual decision making.

For invasive pulmonary aspergillosis, L‐AMB was the preferred first‐line agent in 71.0% of centres, followed by voriconazole (37.1%) (Figure 4B). Fourteen centres reported more than one preferred first‐line agent, and 24 centres more than one alternative agent. Combination antifungal therapy was chosen as the preferred first‐line option only in centres with established SOPs for antifungal treatment (19.1% vs. 0.0% in centres without antifungal treatment SOPs; *p* = 0.098).

In‐house therapeutic drug monitoring (TDM) of voriconazole was available in 51.6% of the centres but was less frequently available for posaconazole (38.7%) and isavuconazole (19.4%).

The German National Reference Center for Invasive Fungal Infections, affiliated with the Leibniz Institute for Natural Product Research and Infection Biology, Hans Knöll Institute (Leibniz‐HKI) in Jena, was known to 72.6% of centres, with centres from Austria (1/5) and Germany (24/39, 61.5%) utilising it, mostly for expert species identification and in vitro susceptibility testing.

**FIGURE 5:**
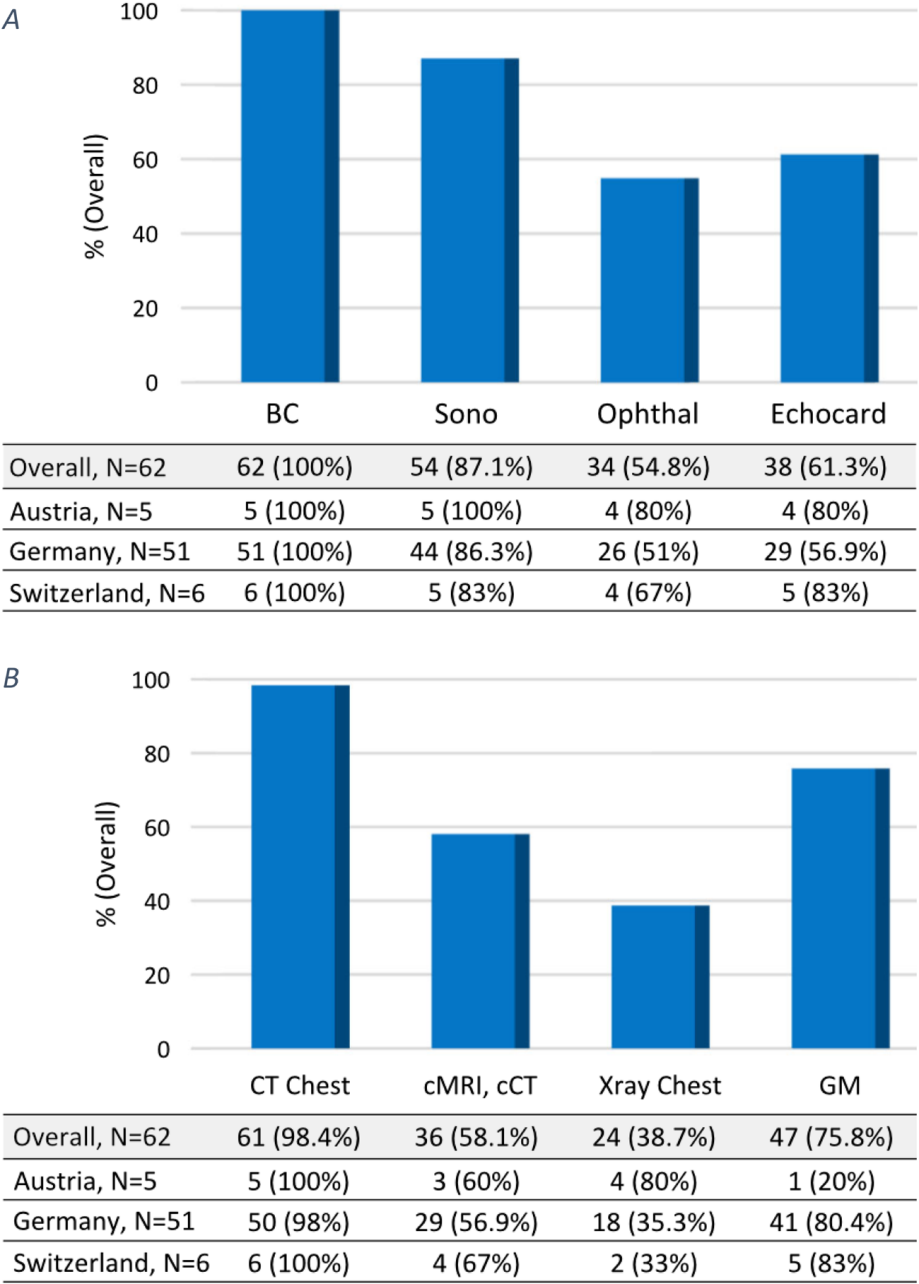
Follow‐up strategies for candidaemia and invasive pulmonary aspergillosis in 62 paediatric cancer centres. (A) Candidaemia. BC, blood culture; Echocard, echocardiography; Ophthal, ophthalmoscopy, Sono, sonography abdomen. (B) Invasive pulmonary aspergillosis. cCT, cranial computed tomography, GM, galactomannan, MRI, magnetic resonance imaging.

Follow‐up in cases of candidaemia included routine blood cultures at all centres, and abdominal sonography in 87.1% (54/62) to assess organ involvement (Figure 5). Echocardiography and ophthalmoscopy were performed in 54.8% and 61.3% of centres, respectively. The use of ophthalmoscopy and echocardiography during follow‐up of candidaemia was not significantly associated with the number of diagnoses per year, the availability of ID consultation services, and ID expertise.

Follow‐up CT of the chest for invasive pulmonary aspergillosis was performed in all but one centre that preferred thorax MRI over CT. Cranial MRI or CT was employed in 58.1% of centres to evaluate for central nervous system involvement. Additionally, galactomannan testing was used in the follow‐up setting of aspergilloses by 75.8% of the centres.

### 3.3 Challenges in IFD Management

Twenty‐five (40.3%) centres identified challenges in the management of IFDs. These centres reported limitations in IFD awareness, difficulties in managing drug–drug interactions between antifungal agents and chemotherapeutics, delays and complexity in interpreting fungal biomarkers (often due to reliance on external laboratories), and prolonged turnaround times for TDM. Box 1 provides an overview of these limitations and outlines targeted strategies to address them, including enhanced training programmes, the development of decision‐making tools and standardised guidelines, and practical recommendations for managing drug interactions. In addition, regular joint clinical rounds and case discussions can strengthen interdisciplinary collaboration between oncology and ID specialists, ultimately improving both the diagnosis and therapeutic outcomes of IFD.

BOX 1 **Challenges in Managing Fungal Infections in Paediatric Cancer Centres and Targeted Strategies for Improvement.**

**Table:** 

Challenges	Targeted strategies
Limited awareness and training due to low incidence of IFDs and limited experience	Provide education and training programs Develop decision‐making tools and standardised guidelines
Drug–drug interactions between antifungal agents and chemotherapeutics, impacting efficacy and increasing the risk for breakthrough IFD	Create practical guidelines for managing such interactions Promote use of antifungal agents that have fewer interactions Utilise in‐house expertise from pharmacists
Delays and complexity in interpreting fungal biomarkers, especially with external laboratories	Standardise testing protocols to avoid excessive or unnecessary tests Improve in‐house diagnostic capacity Train medical staff in interpreting fungal biomarkers Use point‐of‐care tests
Delays in TDM, often requiring external laboratories	Establish in‐house TDM capabilities for key antifungal agents Adopt rapid testing platforms

Abbreviations: IFD, invasive fungal disease; TDM, therapeutic drug monitoring.

## 4 Discussion

This study, which is the first comprehensive analysis of how paediatric oncology centres in German‐speaking countries are logistically equipped, and how they prevent, diagnose and manage IFDs, demonstrates differences in antifungal prophylaxis, diagnostic capabilities and treatment strategies. Most centres have antimicrobial stewardship programmes and ID experts, and more than half have dedicated paediatric ID consultation services. In Germany and Austria, paediatric ID has not been recognised as an independent specialty yet.

Membership in national and less frequently in international ID societies was reported by one‐third of the centres, reflecting an interest in the latest developments and guidelines in ID, which is crucial for patient management and effective antifungal stewardship. National societies often have a more localised focus and can be more accessible for individuals and organisations within a specific country; therefore, it is not surprising that membership in national ID societies was more common than in international societies. Antifungal stewardship and ID experts play a critical role in providing structured guidance on antifungal therapy and training physicians. The added value of close collaboration between ID specialists and oncology teams for patient care, especially in complex fungal infection cases, is well known [28]. Therefore, the involvement of both paediatric oncologists and ID specialists is crucial for the optimal management of immunocompromised patients. Furthermore, integrated training programmes that combine both disciplines may prove beneficial in the future.

Centres that manage more paediatric oncological patients per year were more likely to have in‐house access to advanced diagnostic tools, had established paediatric ID consultation services, and held regular interdisciplinary meetings to discuss patients. This aligns with findings from previous studies suggesting larger institutions are comprehensively equipped due to higher patient volumes and better medical infrastructure [29]. Centres with fewer patients often relied on external laboratories, with potentially longer turn‐around times until results become available that may or may not lead to suboptimal treatment decisions. Early diagnosis and tailored treatment strategies are associated with better outcomes in patients with IFDs; thus, a high level of suspicion for IFDs in addition to sufficient infrastructure is important to direct diagnostics and antifungal treatment effectively [30].

Standard diagnostic methods, such as microscopy, culture and histopathology, were available in almost all centres, whereas more advanced diagnostic tools like NGS, galactomannan and β‐D‐glucan testing were less commonly available. A recent Austrian survey also demonstrated considerable inter‐institutional variation in access to molecular and antigen assays, even among tertiary centres [31]. Centres with established ID consultation services were more likely to utilise galactomannan testing, potentially indicating the critical interplay between clinical expertise and diagnostic capabilities. Interpreting galactomannan indices requires careful consideration of local epidemiology, clinical history and symptoms, necessitating advanced expertise and preferably an interdisciplinary team to guide treatment decisions [32, 33, 34].

Diagnostic infrastructure in paediatric cancer centres faces several limitations. Radiologists, preferentially with a specialised training in paediatric patients and paediatric oncologists must have high expertise in diagnosing and managing paediatric IFD to minimise misdiagnosis. Additionally, although MRI is theoretically available around the clock, the need for sedation in paediatric patients may restrict access due to potential limited availability of anaesthesiologists. Such restrictions can lead to delays in diagnosis but also in treatment decisions being made without sufficient imaging information, increasing the risk for undertreatment in these vulnerable patients.

The reported median cumulative IFD incidence of 4.6% is relatively high, and most likely reflects the high percentage of leukaemia patients with and without HCT who are at an increased risk for IFD but might still be underestimated. Prospective data from six hospitals in Chile, including 777 high‐risk febrile neutropenia episodes in children with cancer, revealed varying IFD frequencies across different cancer types and an overall rise over time. Among the 257 persistent high‐risk febrile neutropenia episodes, IFD incidence increased from 8.5% (95% CI: 5.2–13.5; 8.7 per 1000 neutropenia‐days) in 2004–2006 to 14.6% (95% CI: 10.5–19.9; 13.6 per 1000 neutropenia‐days) in 2016–2020 [35]. Evaluating how specific resources impact IFD incidence is challenging because of multiple confounding factors, including the local epidemiology, patient demographics, antifungal prophylaxis use, heterogeneity in diagnostic practices, expertise and awareness levels, infrastructure quality, personnel workload, and potentially also recall and reporting bias.

Our survey identified heterogeneous approaches to antifungal prophylaxis and treatment across centres. L‐AMB (BII recommendation [3]) was the most commonly used prophylactic antifungal, likely favoured for its broad‐spectrum activity and absence of a relevant drug–drug interaction profile compared to azoles. However, the need for slow intravenous administration and the lack of legal authorisation for this indication are disadvantages. Azoles, while effective against moulds, should be used with caution, as they can lead to significant drug–drug interactions with chemotherapeutic drugs like vincristine, cyclophosphamide and methotrexate, which may be associated with significantly more side effects [36, 37]. In a recent cohort study among patients with ALL, posaconazole as prophylaxis was linked to a 93% lower risk of adverse events (HR: 0.07, *p* < 0.001) compared with L‐AmB, while maintaining comparable rates of breakthrough fungal infections in both high‐ and low‐risk groups [38]. Evidence on the optimal antifungal prophylaxis for distinct patient subgroups, including non‐high‐risk ALL patients, is limited, resulting in varied clinical practices and a lack of universal standardised prophylaxis guidelines [39].

For candidaemia, centres predominantly preferred L‐AMB (AII recommendation [3]) and echinocandins (AII [3]) as first‐line therapies, despite the comparable efficacy of both drug classes but a superior safety profile of echinocandins [40]. It is important to note that the paed‐EQUAL Score for candidaemia was not evaluated in this study, as its development was conducted in parallel with our investigation [14].

Invasive aspergillosis was primarily managed using L‐AMB (BII recommendation [3]), followed by voriconazole (AII), despite the unfavourable drug–drug interactions between azoles and chemotherapeutics [36, 37].

Many centres have to rely on external services for TDM, for example, commonly for isavuconazole and posaconazole, that may lead to delay in treatment adjustments required for optimising trough levels for activity or to avoid toxicity [41]. TDM is particularly important for voriconazole due to the compound's high intra‐ and interindividual pharmacokinetic variability, and exposure‐dependent toxicities [42]. Faster turnaround times can be achieved with more in‐house capabilities and standardised approaches, especially where on‐site tools are limited.

Participation in mycology‐related research and clinical trials was limited, particularly among smaller centres. This may reflect the challenges of conducting paediatric trials with particularly stringent regulations for children, the complexity of IFDs involving various pathogens and indications, and the already small population of paediatric patients affected by IFDs. Strengthening collaboration between centres and standardising data collection could facilitate multicentre studies. Further investment in continuous training in the ID supportive care and IFD management in paediatric patients and fostering interregional collaboration among experts could address gaps in knowledge and practice, ultimately improving patient outcomes.

Several limitations of this fixed‐response survey must be acknowledged. Centre‐level self‐reporting may have introduced a reporting bias as we did not verify against patient‐level data. Thus, intra‐centre variability or actual adherence to reported strategies could not be accounted for. Key terms likely varied in interpretation across centres, for example, ID consultation service that may span everything from informal phone advice to formal multidisciplinary rounds, and availability of SOPs which can range from detailed, regularly updated guidelines to general checklists, which make comparisons between infrastructure and actual clinical behaviour complicated. We acknowledge that the absence of a detectable association between the reported cumulative IFD incidence and availability of SOPs for antifungal prophylaxis or availability of ID consultation service in our data may not reflect a true lack of protective effect of the latter but may be considered artefacts of reporting and diagnostic complexity in paediatric oncology. Furthermore, SOP implementation and established ID consultation services may drive more rigorous case finding due to screening and potentially lower diagnostic thresholds, which could paradoxically lead to more IFD cases in respective centres despite benefiting clinically from prophylaxis and expert review. On the contrary, centres with fewer resources may under‐detect IFD which would in turn mask any positive effect of prophylaxis protocols or additional involvement of ID specialists. The pre‐defined options for prophylaxis, diagnostics, and treatment pathways focused on pulmonary aspergillosis and candidaemia as the most frequent paediatric IFDs, so we could not assess approaches to less common IFDs like mucormycosis or fusariosis. Due to the clinical complexity of paediatric IFD and the broader scope of our questionnaire, in‐depth analyses in specific patient populations remain an important goal for future studies. Collecting patient‐level data in future studies would also then allow for linking centre‐level capabilities to clinical endpoints such as time to diagnosis or IFD‐related mortality.

To the best of our knowledge, this is the first study aiming at systematically mapping current practices in the prevention, diagnosis and management of IFDs in paediatric cancer centres across German‐speaking countries. With the high response rate, we were able to provide a representative picture on institutional and regional approaches in Germany, Austria and Switzerland. Our work highlights critical gaps, for example, in diagnostic availability and antifungal drug monitoring, where harmonisation across centres could significantly reduce variability in care and improve outcomes. Addressing these gaps will require significant investment in infrastructure, training and personnel, as well as strengthening interregional collaborations between experts. Furthermore, investing in robust digital infrastructure and fostering networks between healthcare providers and specialists would deliver the expertise directly to patients regardless of their location, ensuring standardised and optimised patient management. This overview may serve as a starting point for the development of a strategic roadmap toward future paediatric antifungal stewardship programmes.

## Author Contributions

**Danila Seidel:** conceptualization, methodology, data curation, formal analysis, visualization, writing – original draft, investigation, project administration, validation, supervision. **Alexander Claviez:** investigation, writing – review and editing. **Georg C. Schwabe:** writing – review and editing, investigation. **Marius Rohde:** writing – review and editing, investigation. **Martina Stiefel:** investigation, writing – review and editing. **Michael Frühwald:** investigation, writing – review and editing. **Michaela Döring:** investigation, writing – review and editing. **Michaela Nathrath:** investigation, writing – review and editing. **Udo Kontny:** investigation, writing – review and editing. **Andreas H. Groll:** writing – original draft, methodology, conceptualization, supervision, investigation. **Thomas Lehrnbecher:** supervision, methodology, writing – original draft, conceptualization, investigation. **Zoi Dorothea Pana:** methodology, conceptualization, writing – original draft. **Daniel Ebrahimi‐Fakhari:** writing – review and editing, investigation. **Sarina K. Butzer:** investigation, writing – review and editing. **Katrin Mehler:** writing – review and editing, investigation. **Ilana Reinhold:** conceptualization, methodology, writing – review and editing. **Arne Simon:** investigation, writing – review and editing. **Christian Dohna‐Schwake:** investigation, writing – review and editing. **Ines Mack:** writing – review and editing, investigation. **Nicole Bodmer:** writing – review and editing, investigation. **Tim Niehues:** writing – review and editing, investigation. **Alfred Längler:** investigation, writing – review and editing. **Alfred Leipold:** investigation, writing – review and editing. **Aram Prokop:** investigation, writing – review and editing. **Bastian Brummel:** investigation, writing – review and editing. **Beate Winkler:** writing – review and editing, investigation. **Bernd Gruhn:** investigation, writing – review and editing. **Carl Friedrich Classen:** investigation, writing – review and editing. **Carsten Friedrich:** investigation, writing – review and editing. **Christa Koenig:** investigation, writing – review and editing. **Christian Flotho:** investigation, writing – review and editing. **Fiona Poyer:** investigation, writing – review and editing. **Freimut Schilling:** investigation, writing – review and editing. **Gabriele Calaminus:** investigation, writing – review and editing. **Geeke Sieben:** investigation, writing – review and editing. **Harald Reinhard:** writing – review and editing, investigation. **Heiko‐Manuel Teltschik:** writing – review and editing, investigation. **Heinz Hengartner:** investigation, writing – review and editing. **Jana Stursberg:** writing – review and editing, investigation. **Jeanette Greiner:** writing – review and editing, investigation. **Johann Greil:** investigation, writing – review and editing. **Jörg Leyh:** investigation, writing – review and editing. **Jörn‐Sven Kühl:** writing – review and editing, investigation. **Karoline Ehlert:** writing – review and editing, investigation. **Konrad Bochennek:** writing – review and editing, investigation. **Martin Demmert:** writing – review and editing, investigation. **Matthias Eyrich:** investigation, writing – review and editing. **Meinolf Siepermann:** investigation, writing – review and editing. **Milen Minkov:** investigation, writing – review and editing. **Monika Streiter:** writing – review and editing, investigation. **Neil Jones:** investigation, writing – review and editing. **Nora Naumann‐Bartsch:** investigation, writing – review and editing. **Norbert Jorch:** writing – review and editing, investigation. **Olaf Beck:** writing – review and editing, investigation. **Rita Beier:** writing – review and editing, investigation. **Roman Crazzolara:** investigation, writing – review and editing. **Silke Kietz:** investigation, writing – review and editing. **Simon Vieth:** writing – review and editing, investigation. **Stefan Fröhling:** investigation, writing – review and editing. **Stephan Lobitz:** writing – review and editing, investigation. **Sujal Ghosh:** investigation, writing – review and editing. **Tanja C. Vallée:** writing – review and editing, investigation. **Thilo Müller:** writing – review and editing, investigation. **Thomas Wiesel:** investigation, writing – review and editing. **Tobias Däbritz:** investigation, writing – review and editing. **Uwe Thiel:** investigation, writing – review and editing. **Volker Strenger:** investigation, writing – review and editing. **Wolfgang R. Eberl:** investigation, writing – review and editing. **Oliver A. Cornely:** writing – review and editing, supervision, funding acquisition, resources, investigation.

## Conflicts of Interest

A.H.G. has received grants from Gilead, Merck, Sharp & Dohme and Pfizer and has served as a consultant to Amplyx, Astellas, Basilea, F2G, Gilead, Merck, Sharp & Dohme, Mundipharma, Pfizer and Scynexis. O.A.C. reports grants or contracts from iMi, iHi, DFG, BMBF, Cidara, DZIF, EU‐DG RTD, F2G, Gilead, MedPace, MSD, Mundipharma, Octapharma, Pfizer, Scynexis; consulting fees from Abbvie, AiCuris, Basilea, Biocon, Boston Strategic Partners, Cidara, Elion Therapeutics, Gilead, GSK, IQVIA, Janssen, Matinas, MedPace, Menarini, Melinta, Molecular Partners, MSG‐ERC, Mundipharma, Noxxon, Octapharma, Pardes, Partner Therapeutics, Pfizer, PSI, Scynexis, Seres, Seqirus, Shionogi, The Prime Meridian Group; speaker and lecture honoraria from Abbott, Abbvie, Akademie für Infektionsmedizin, Al‐Jazeera Pharmaceuticals/Hikma, amedes, AstraZeneca, Deutscher Ärzteverlag, Gilead, GSK, Grupo Biotoscana/United Medical/Knight, Ipsen Pharma, Medscape/WebMD, MedUpdate, MSD, Moderna, Mundipharma, Noscendo, Paul‐Martini‐Stiftung, Pfizer, Sandoz, Seqirus, Shionogi, streamedup!, Touch Independent, Vitis; payment for expert testimony from Cidara; participation on a DRC, DSMB, DMC, Advisory Board for AstraZeneca, Cidara, IQVIA, Janssen, MedPace, Melinta, PSI, Pulmocide, Vedanta Biosciences. Other authors had no conflicts of interest. T.N. has received authorship fees from uptodate.com (Wellesley, Massachusetts, USA) and reimbursement of travel expenses during consultancy work for the European Medicines Agency (EMA), steering committees of the PENTA Paediatric European Network for Treatment of AIDS (Padua, Italy), the Juvenile Inflammatory Cohort (JIR) (Lausanne, Switzerland). U.T. is funded by the Deutsche Forschungsgemeinschaft (DFG, German Research Foundation)—Project number 501830041. T.L. served as a consultant to Gilead Sciences, Pfizer, Merck/MSD, Mundipharma, Roche, Recordati, GlaxoSmithKline and Pharming, and in the speaker's bureau of Gilead Sciences, Merck/MSD, AstraZeneca, Recordati, Pfizer, Mundipharma and Sanofi Pasteur. V.S. served as a consultant to Merck/MSD, Pfizer and SANOFI, and has received speaker honoraria from GlaxoSmithKline, Merck/MSD, Pfizer and SANOFI.

## Supporting information


Table S1.

## Data Availability

The data that support the findings of this study are available on request from the corresponding author. The data are not publicly available due to privacy or ethical restrictions.
