# Supplementary material for: Approaches to Invasive Fungal Diseases in Paediatric Cancer Centres: An Analysis of Current Practices and Challenges in Germany, Austria and Switzerland
Source: Mycoses. 2025 Jun 14;68(6):e70074. doi: 10.1111/myc.70074 (PMC12166348; doi:10.1111/myc.70074)
Supplement: Supplementary file 1 — Table S1. [file MYC-68-e70074-s001.docx]

# Supplementary Information

Supplementary Table 1 Questionnaire in German language as provided to the pediatric physicians (left) and English translation (right)

| **Deutsch** | |  | **English** | | |
| --- | --- | --- | --- | --- | --- |
| **Behandlung von Pilzinfektionen in Kindern** | |  | **Management of Invasive Fungal Infections in Pediatric Patients** | | |
|  |  |  |  |  |  |
| **Name und Einrichtung** |  |  | **Name and Institution** |  |  |
|  |  |  |  |  |  |
| **Frage** | **Antwortoptionen** |  | **Question** | **Response Options** |  |
| Ihr Vor- und Nachname | Freitext |  | Your First and Last Name | Free text |  |
| Krankenhaus/Einrichtung | Freitext |  | Hospital/Institution | Free text |  |
| Stadt | Freitext |  | City | Free text |  |
| E-Mail-Adresse | Freitext |  | Email Address | Free text |  |
| Name und Kontakt des Autors, wenn abweichend von oben | Freitext |  | Name and Contact of the Author, if different from above | Free text |  |
|  |  |  |  |  |  |
| **Ihre Position** |  |  | **Your Position** |  |  |
|  |  |  |  |  |  |
| **Frage** | **Antwortoptionen** |  | **Question** | **Response Options** |  |
| Ihre Position | - Chefärztin \| Chefarzt - Oberärztin \| Oberarzt - Fachärztin \| Facharzt mit Schwerpunktbezeichnung |  | Your Position | - Chief Physician - Senior Physician - Specialist with additional training in pediatric ID |  |
|  |  |  |  |  |  |
| **Zusatzweiterbildung Infektiologie** | |  | **Additional Training in Infectious Diseases** | | |
|  |  |  |  |  |  |
| **Frage** | **Antwortoptionen** |  | **Question** | **Response Options** |  |
| Hat eine Ärztin oder ein Arzt in Ihrer Kinderklinik die Zusatzweiterbildung Infektiologie? | Ja, Nein |  | Does a physician in your pediatric clinic have additional training in ID? | Yes, No |  |
| Hat eine Ärztin oder ein Arzt in Ihrer kinder-onkologischen Abteilung die Zusatzweiterbildung Infektiologie? | Ja, Nein |  | Does a physician in your pediatric oncology department have additional training in ID? | Yes, No |  |
|  |  |  |  |  |  |
| **Mitgliedschaft in Mykologischen/Infektiologischen Gesellschaften** | |  | **Membership in Mycological/Infectiological Societies** | | |
|  |  |  |  |  |  |
| **Frage** | **Antwortoptionen** |  | **Question** | **Response Options** |  |
| Sind Sie oder KollegInnen aus der kinder-onkologischen Abteilung Mitglied in einer oder mehrerer Mykologischen/Infektiologischen Gesellschaft(en)? | - DGPI (Deutsche Gesellschaft Pädiatrische Infektiologie) - DMykG (Deutschsprachige Mykologische Gesellschaft) - ECMM (European Confederation of Medical Mycology) - ESCMID (European Society of Clinical Microbiology and Infectious Diseases) - ESPID (European Society for Paediatric Infectious Diseases) - IDSA (Infectious Diseases Society of America) - ISHAM (The International Society for Human & Animal Mycology) - MSGERC (Mycoses Study Group) - PEG (Paul-Ehrlich-Gesellschaft für Infektionstherapie) - Andere (Freitext) |  | Are you or colleagues from the pediatric oncology department members of one or more Mycological/ID societies? | - DGPI (Deutsche Gesellschaft Pädiatrische Infektiologie) - DMykG (Deutschsprachige Mykologische Gesellschaft) - ECMM (European Confederation of Medical Mycology) - ESCMID (European Society of Clinical Microbiology and Infectious Diseases) - ESPID (European Society for Paediatric Infectious Diseases) - IDSA (Infectious Diseases Society of America) - ISHAM (The International Society for Human & Animal Mycology) - MSGERC (Mycoses Study Group) - PEG (Paul-Ehrlich-Gesellschaft für Infektionstherapie) - Others (Free text) |  |
|  |  |  |  |  |  |
| **Kinderklinik** | |  | **Pediatric Clinic** | | |
|  |  |  |  |  |  |
| **Frage** | **Antwortoptionen** |  | **Question** | **Response Options** |  |
| Eigene Abteilung Infektiologie in Ihrer Kinderklinik? | Ja, Nein |  | Own ID Department in your pediatric clinic? | Yes, No |  |
|  |  |  |  |  |  |
| Gibt es ein ABS Team/ExpertIn in Ihrer Kinderklinik? | Ja, Nein |  | Is there an ABS Team/Expert in your pediatric clinic? | Yes, No |  |
| Gibt es (zusätzlich zum ABS Team/ExpertIn, wenn zutreffend) einen pädiatrisch-infektiologischen Konsiliardienst in Ihrer Kinderklinik? | Ja, Nein |  | Is there (in addition to the ABS Team/Expert, if applicable) a pediatric ID consultation service in your pediatric clinic? | Yes, No |  |
| Erreichbarkeit des pädiatrisch-infektiologischen Konsiliardienstes in Ihrer Kinderklinik | - 5 Tage / tagsüber - 7 Tage / 24 h - Kein eigener Konsiliardienst |  | Availability of the pediatric ID consultation service in your pediatric clinic | - 5 days/daytime - 7 days/24 hours - No own consultation service |  |
| Gibt es eine regelmäßige fachübergreifende infektiologische Konferenz in Ihrer Kinderklinik? | Ja, Nein |  | Is there a regular interdisciplinary ID conference in your pediatric clinic? | Yes, No |  |
| Teilnahme an klinischen Studien zu mykologischen Themen seit 2019 (interventionell, nicht-interventionell) | Ja, Nein |  | Participation in clinical studies on mycological topics since 2019 (interventional, non-interventional) | Yes, No |  |
| Teilnahme an Zulassungsstudien von antimykotischen Substanzen seit 2019 | Ja, Nein |  | Participation in approval studies of antifungal substances since 2019 | Yes, No |  |
| Publikationen zu mykologischen Themen aus der kinder-onkologischen Abteilung seit 2019 | - >5 Publikationen - ≤5 Publikationen - Keine Publikation zum Thema |  | Publications on mycological topics from the pediatric oncology department since 2019 | - >5 Publications - ≤5 Publications - No Publications on the topic |  |
|  |  |  |  |  |  |
| **Daten zur pädiatrischen Onkologie und Stammzelltransplantation und Richtlinien zur Behandlung von Pilzinfektionen** | |  | **Data on Pediatric Oncology and Stem Cell Transplantation and Guidelines for the Treatment of Fungal Infections** | | |
|  |  |  |  |  |  |
| **Frage** | **Antwortoptionen** |  | **Question** | **Response Options** |  |
| Pädiatrische onkologische Neudiagnosen in Ihrer Kinderklinik pro Jahr | Anzahl: Neudiagnosen im Jahr 2023; bitte schätzen, falls nicht bekannt |  | Pediatric oncological new diagnoses in your pediatric clinic per year | Number: New diagnoses in 2023; please estimate if not known |  |
| Pädiatrische allogene hämatopoetische Zelltransplantations-Fälle in Ihrer Kinderklinik pro Jahr | Anzahl: allogene SZT in Kindern im Jahr 2023; bitte schätzen, falls nicht bekannt |  | Pediatric allogeneic cell transplantation cases in your pediatric clinic per year | Number: Allogeneic SCT in children in 2023; please estimate if not known |  |
| Pädiatrische onkologische PatientInnen mit einer Pilzinfektion in Ihrer Kinderklinik pro Jahr (gesicherte (proven) oder wahrscheinliche (probable) Pilzinfektionen nach EORTC/MSG Kriterien - verlangt den Nachweis des Erregers mittels Kultur, PCR oder Antigen)) | Anzahl:  Kinder im Jahr 2023; bitte schätzen, falls nicht bekannt |  | Pediatric oncological patients with a fungal infection in your pediatric clinic per year (proven or probable fungal infections according to EORTC/MSG criteria - requires evidence of the pathogen via culture, PCR, or antigen) | Number: Children in 2023; please estimate if not known |  |
|  |  |  |  |  |  |
| **Standardarbeitsanweisungen (SOPs)** | |  | **Standard Operating Procedures (SOPs)** | | |
|  |  |  |  |  |  |
| **Frage** | **Antwortoptionen** |  | **Question** | **Response Options** |  |
| Standardarbeitsanweisungen (SOPs) für Prophylaxe, Diagnose und Behandlung von Pilzinfektionen bei Kindern vorhanden in Ihrer Kinderklinik? | - Prophylaxe: Ja, Nein - Diagnose: Ja, Nein - Behandlung: Ja, Nein |  | Are Standard Operating Procedures (SOPs) for prophylaxis, diagnosis, and treatment of fungal infections in children available in your pediatric clinic? | - Prophylaxis: Yes, No - Diagnosis: Yes, No - Treatment: Yes, No |  |
|  |  |  |  |  |  |
| **Leitlinien und Prävention** | |  | **Guidelines and Prevention** | | |
|  |  |  |  |  |  |
| **Frage** | **Antwortoptionen** |  | **Question** | **Response Options** |  |
| Welchen nationalen oder internationalen Leitlinien folgen Sie bei der Behandlung von Pilzinfektionen bei Kindern? | - AWMF Leitlinien (Arbeitsgemeinschaft der Wissenschaftlichen Medizinischen Fachgesellschaften, [www.awmf.org](http://www.awmf.org)) - ECIL-8 (8th European Conference on Infections in Leukaemia (Groll et al, 2021) - Guidelines for the diagnosis, prevention, and treatment of invasive fungal diseases in paediatric patients with cancer or post-haematopoietic cell transplantation) - Guideline for the Management of Fever and Neutropenia in Pediatric Patients With Cancer and Hematopoietic Cell Transplantation Recipients: 2023 Update (Lehrnbecher et al, 2023, J Clin Oncol) - ESCMID / ECMM / ISHAM Guidelines (Candida, Cornely et al, CMI 2012 // Rare Yeast infections, Arendrup et al, CMI 2014 // Mucormycosis, Cornely et al, Lancet Infect Dis 2019 // Rare yeast infections, Chen et al, Lancet Infect Dis 2021 // Rare mold infections, Hoenigl et al, The Lancet 2021) [www.ecmm.info/guidelines/](http://www.ecmm.info/guidelines/) - IDSA Clinical Practice Guideline (Candidiasis, Pappas et al, CID 2016 // Aspergillosis, Patterson et al, CID 2016) - Andere (Freitext) |  | Which national or international guidelines do you follow in the treatment of fungal infections in children? | - AWMF Guidelines (Association of the Scientific Medical Societies in Germany, [www.awmf.org](http://www.awmf.org)) - ECIL-8 (8th European Conference on Infections in Leukaemia (Groll et al., 2021) - Guidelines for the diagnosis, prevention, and treatment of invasive fungal diseases in pediatric patients with cancer or post-hematopoietic cell transplantation) - Guideline for the Management of Fever and Neutropenia in Pediatric Patients With Cancer and Hematopoietic Cell Transplantation Recipients: 2023 Update (Lehrnbecher et al., 2023, J Clin Oncol) - ESCMID/ECMM/ISHAM Guidelines (Candida, Cornely et al., CMI 2012 // Rare Yeast infections, Arendrup et al., CMI 2014 // Mucormycosis, Cornely et al., Lancet Infect Dis 2019 // Rare yeast infections, Chen et al., Lancet Infect Dis 2021 // Rare mold infections, Hoenigl et al., The Lancet 2021) [www.ecmm.info/guidelines/](http://www.ecmm.info/guidelines/) - IDSA Clinical Practice Guideline (Candidiasis, Pappas et al., CID 2016 // Aspergillosis, Patterson et al., CID 2016) - Others (Free text) |  |
|  |  |  |  |  |  |
| **ECMM EQUAL Scores bekannt?** | |  | **ECMM EQUAL Scores Known?** | | |
|  |  |  |  |  |  |
| **Frage** | **Antwortoptionen** |  | **Question** | **Response Options** |  |
| ECMM EQUAL Scores bekannt? | Bekannt, Nicht bekannt |  | Are ECMM EQUAL Scores known? | Known, Not known |  |
|  |  |  |  |  |  |
| **Verfügbarkeit von Ressourcen und Leitlinienadhärenz** | |  | **Availability of Resources and Adherence to Guidelines** | | |
|  |  |  |  |  |  |
| **Mikrobiologie und Pathologie** | |  | **Microbiology and Pathology** | | |
|  |  |  |  |  |  |
| **Frage** | **Antwortoptionen** |  | **Question** | **Response Options** |  |
| Welche Methoden stehen Ihnen im Allgemeinen in Ihrem Haus zur Pilz-Diagnostik zur Verfügung? | - Direkte Mikroskopie - Kultur - Empfindlichkeitstestung - Galaktomannan - β-D-Glukan - Cryptococcus-Antigen-Test - PCR-Verfahren - Next Generation Sequenzierung - Histopathologie - Andere (Freitext) |  | Which methods are generally available in your facility for fungal diagnostics? | - Direct Microscopy - Culture - Sensitivity Testing - Galactomannan - β-D-Glucan - Cryptococcus Antigen Test - PCR Methods - Next Generation Sequencing - Histopathology - Others (Free text) |  |
| Wird bei invasiven Isolaten eine Empfindlichkeitstestung durchgeführt? | - Ja, immer - Ja, bei bestimmten Pilzen (bitte benennen) oder v.A.   Resistenzen   - Nein |  | Is sensitivity testing performed on invasive isolates? | - Always - Yes, for certain fungi (please specify) or potential resistances - No |  |
|  |  |  |  |  |  |
| **Nationales Referenzzentrum für Mykologie (NRZMyk), Deutschland** | |  | **National Reference Center for Mycology (NRZMyk), Germany** | | |
|  |  |  |  |  |  |
| **Frage** | **Antwortoptionen** |  | **Question** | **Response Options** |  |
| Nationales Referenzzentrum für Mykologie (NRZMyk) bekannt? | Bekannt, Nicht bekannt |  | Is the National Reference Center for Mycology (NRZMyk) known? | Known, Not known |  |
| NRZMyk bereits konsultiert? | Ja, Nein |  | Have you already consulted NRZMyk? | Yes, No |  |
| Gründe, warum das NRZMyk konsultiert wurde: | - Durchführung und Interpretation von Resistenztests - Zur Speziesidentifikation bei Verdacht auf seltene oder ungewöhnliche Pilzinfektionen - Beratung zu prophylaktischen Maßnahmen - Beratung zu spezifischen antifungalen Therapieoptionen, insbesondere bei komplizierten oder resistenten Infektionen - Beratung zu Maßnahmen zur Eindämmung bei gehäuften Infektionsfällen oder Ausbrüchen - Spezielle Untersuchungen wie Molekularbiologie oder genetische Analysen von Pilzen - Daten für epidemiologische Studien bereitstellen - Andere (Freitext) |  | Reasons for consulting NRZMyk: | - Conducting and interpreting resistance tests - Species identification in case of suspected rare or unusual fungal infections - Advise on prophylactic measures - Advise on specific antifungal therapy options, especially in complicated or resistant infections - Advise on measures to contain frequent infection cases or outbreaks - Special investigations such as molecular biology or genetic analyses of fungi - Providing data for epidemiological studies - Others (Free text) |  |
|  |  |  |  |  |  |
| **Bildgebung und invasive Verfahren** | |  | **Imaging and Invasive Procedures** | | |
|  |  |  |  |  |  |
| **Frage** | **Antwortoptionen** |  | **Question** | **Response Options** |  |
| Welche bildgebenden und endoskopischen Methoden stehen Ihnen im Allgemeinen in Ihrem Haus zur Verfügung? | - CT - MRT - Röntgenuntersuchung - PET/CT, PET/MRI - Sonographie - Bronchoskopie - Bildunterstützte Biopsien (CT oder ultraschall-gesteuert) |  | Which imaging and endoscopic methods are generally available in your facility? | - CT - MRI - X-ray - PET/CT, PET/MRI - Ultrasound - Bronchoscopy - Image-guided biopsies (CT or sonographic) |  |
| CT Verfügbarkeit | - CT-Untersuchungen sind 24/7 verfügbar - Nein, CT-Untersuchungen sind nicht 24/7 verfügbar |  | CT Availability | - CT examinations available 24/7 - No, CT examinations not available 24/7 |  |
| MRT Verfügbarkeit | - MRT-Untersuchungen sind 24/7 verfügbar - Nein, MRT-Untersuchungen sind nicht 24/7 verfügbar |  | MRI Availability | - MRI examinations available 24/7 - No, MRI examinations not available 24/7 |  |
| Therapeutic Drug Monitoring vorhanden? | - Voriconazol: Extern, In house - Posaconazol: Extern, In house - Isavuconazol: Extern, In house |  | Therapeutic Drug Monitoring Available? | - Voriconazole: External, In-house - Posaconazole: External, In-house - Isavuconazole: External, In-house |  |
|  |  |  |  |  |  |
| **Antifungale Prophylaxe der ersten Wahl in der kinder-onkologischen Abteilung** | |  | **Antifungal Prophylaxis of First Choice in the Pediatric Oncology Department** | | |
|  |  |  |  |  |  |
| **Frage** | **Antwortoptionen** |  | **Question** | **Response Options** |  |
| Antifungale Prophylaxe der ersten Wahl in der kinder-onkologischen Abteilung für jeweils:   - AML (Akute myeloische Leukämie) - Hochrisiko-ALL (Akute lymphatische Leukämie) - Rezidivierende akute Leukämie - Allogene SZT vor Engraftment - Graft-versus-Host-Disease- und augmentierte Immunsuppression | - Fluconazol - Itraconazol - Posaconazol - Voriconazole - Amphotericin B - Caspofungin - Micafungin - Keine Prophylaxe - Nicht zutreffend |  | Antifungal prophylaxis of first choice in the pediatric oncology department for each:   - AML (Acute Myeloid Leukemia) - High-risk ALL (Acute Lymphoblastic Leukemia) - Recurrent Acute Leukemia - Allogeneic SCT before Engraftment - Graft-versus-Host Disease and Augmented Immunosuppression | - Fluconazole - Itraconazole - Posaconazole - Voriconazole - Amphotericin B - Caspofungin - Micafungin - No Prophylaxis - Not Applicable |  |
|  |  |  |  |  |  |
| **Was ist die bevorzugte Strategie in der kinder-onkologischen Abteilung** | |  | **Preferred Strategy in the Pediatric Oncology Department** | | |
|  |  |  |  |  |  |
| **Frage** | **Antwortoptionen** |  | **Question** | **Response Options** |  |
| Was ist die bevorzugte Strategie in der kinder-onkologischen Abteilung? | - Empirische antifungale Therapie (basierend auf persistierendem Fieber trotz Antibiotika in Neutropenie) - Präemptive antifungale Therapie (basierend auf Bildgebung und/oder Biomarkern) |  | What is the preferred strategy in the pediatric oncology department? | - Empirical Antifungal Therapy (based on persistent fever despite antibiotics in neutropenia) - Preemptive Antifungal Therapy (based on imaging and/or biomarkers) |  |
|  |  |  |  |  |  |
| **Candidämie: Erstlinientherapie und alternative Erstlinientherapie bei Kindern in Ihrer Einrichtung (systemische antifungale Therapie)** | |  | **Candidemia: First-line and Alternative First-line Therapy in Your Facility (Systemic Antifungal Therapy)** | | |
|  |  |  |  |  |  |
| **Frage** | **Antwortoptionen** |  | **Question** | **Response Options** |  |
| Candidämie: Erstlinientherapie und alternative Erstlinientherapie bei Kindern in Ihrer Einrichtung | - Echinocandin (Erstlinie), Echinocandin (Alternative zur Erstlinie) - Fluconazol (Erstlinie), Fluconazol (Alternative zur Erstlinie) - Voriconazol (Erstlinie), Voriconazol (Alternative zur Erstlinie) - Amphotericin B (liposomale Formulierung) (Erstlinie), Amphotericin B (liposomale Formulierung) (Alternative zur Erstlinie) - Kombination (Erstlinie), Kombination (Alternative zur Erstlinie) |  | Candidemia: First-line and alternative first-line therapy in your facility | - Echinocandin (First-line), Echinocandin (Alternative to First-line) - Fluconazole (First-line), Fluconazole (Alternative to First-line) - Voriconazole (First-line), Voriconazole (Alternative to First-line) - Amphotericin B (Liposomal Formulation) (First-line), Amphotericin B (Liposomal Formulation) (Alternative to First-line) - Combination (First-line), Combination (Alternative to First-line) |  |
|  |  |  |  |  |  |
| **Wann beenden Sie die Therapie einer unkomplizierten Candidämie?** | |  | **When Do You Discontinue Therapy for an Uncomplicated Candidemia?** | | |
|  |  |  |  |  |  |
| **Frage** | **Antwortoptionen** |  | **Question** | **Response Options** |  |
| Wann beenden Sie die Therapie einer unkomplizierten Candidämie? | - <14 Tage nach der ersten negativen Blutkultur - ≥14 Tage nach der ersten negativen Blutkultur - Individuell |  | When do you discontinue therapy for an uncomplicated candidemia? | - <14 days after the first negative blood culture - ≥14 days after the first negative blood culture - Individual |  |
|  |  |  |  |  |  |
| **Pulmonale Aspergillose: Erstlinientherapie und alternative Erstlinientherapie bei Kindern in Ihrer Einrichtung** | |  | **Pulmonary Aspergillosis: First-line and Alternative First-line Therapy in Your Facility** | | |
|  |  |  |  |  |  |
| **Frage** | **Antwortoptionen** |  | **Question** | **Response Options** |  |
| Pulmonale Aspergillose: Erstlinientherapie und alternative Erstlinientherapie bei Kindern in Ihrer Einrichtung | - Voriconazole (Erstlinie), Voriconazole (Alternative zur Erstlinie) - Posaconazole (Erstlinie), Posaconazole (Alternative zur Erstlinie) - Isavuconazole (Erstlinie), Isavuconazole (Alternative zur Erstlinie) - Liposomales Amphotericin B (Erstlinie), Liposomales Amphotericin B (Alternative zur Erstlinie) - Echinocandin (Erstlinie), Echinocandin (Alternative zur Erstlinie) - Kombination (Erstlinie), Kombination (Alternative zur Erstlinie) |  | Pulmonary Aspergillosis: First-line and alternative first-line therapy in your facility | - Voriconazole (First-line), Voriconazole (Alternative to First-line) - Posaconazole (First-line), Posaconazole (Alternative to First-line) - Isavuconazole (First-line), Isavuconazole (Alternative to First-line) - Liposomal Amphotericin B (First-line), Liposomal Amphotericin B (Alternative to First-line) - Echinocandin (First-line), Echinocandin (Alternative to First-line) - Combination (First-line), Combination (Alternative to First-line) |  |
|  |  |  |  |  |  |
| **Candidämie: Was sind übliche Verlaufskontrollen in Ihrer Einrichtung?** | |  | **Candidemia: What Are the Usual Follow-up Controls in Your Facility?** | | |
|  |  |  |  |  |  |
| **Frage** | **Antwortoptionen** |  | **Question** | **Response Options** |  |
| Candidämie: Was sind übliche Verlaufskontrollen in Ihrer Einrichtung? | - Blutkulturen - Sonographie Abdomen - Augenuntersuchung - Echokardiographie - Andere (Freitext) |  | Candidemia: What are the usual follow-up controls in your facility? | - Blood Cultures - Abdominal Ultrasound - Ophthalmoscopy - Echocardiography - Others (Free text) |  |
|  |  |  |  |  |  |
| **Pulmonale Aspergillose: Was sind übliche Verlaufskontrollen in Ihrer Einrichtung?** | |  | **Pulmonary Aspergillosis: What Are the Usual Follow-up Controls in Your Facility?** | | |
|  |  |  |  |  |  |
| **Frage** | **Antwortoptionen** |  | **Question** | **Response Options** |  |
| Pulmonale Aspergillose: Was sind übliche Verlaufskontrollen in Ihrer Einrichtung? | - Thorax-CT - Kraniales MRT / Kraniales CT - Röntgen Thorax - Galaktomannan - Andere (Freitext) |  | Pulmonary Aspergillosis: What are the usual follow-up controls in your facility? | - Thorax CT - Cranial MRI / Cranial CT - Chest X-ray - Galactomannan - Others (Free text) |  |
|  |  |  |  |  |  |
| **Herausforderungen oder Barrieren bei der Prävention, Diagnose und Behandlung von Pilzinfektionen in Ihrer Einrichtung** | |  | **Challenges or Barriers in Preventing, Diagnosing, and Treating Fungal Infections in Your Facility** | | |
|  |  |  |  |  |  |
| **Frage** | **Antwortoptionen** |  | **Question** | **Response Options** |  |
| Herausforderungen oder Barrieren bei der Prävention, Diagnose und Behandlung von Pilzinfektionen in Ihrer Einrichtung? (optional) | Freitext |  | Challenges or barriers in preventing, diagnosing, and treating fungal infections in your facility? (optional) | Free text |  |
